# Relationship between Multiple Sclerosis-Associated *IL2RA* Risk Allele Variants and Circulating T Cell Phenotypes in Healthy Genotype-Selected Controls

**DOI:** 10.3390/cells8060634

**Published:** 2019-06-25

**Authors:** Sophie Buhelt, Helle Bach Søndergaard, Annette Oturai, Henrik Ullum, Marina Rode von Essen, Finn Sellebjerg

**Affiliations:** 1 Danish Multiple Sclerosis Center, Department of Neurology, Copenhagen University Hospital, Rigshospitalet, 2600 Glostrup, Denmark; hbs@rh.dk (H.B.S.); abo@rh.dk (A.O.); marina.rode.von.essen@regionh.dk (M.R.v.E.); finn.thorup.sellebjerg@regionh.dk (F.S.); 2 Department of Clinical Immunology, Center of Clinical Investigation, Copenhagen University Hospital, Rigshospitalet, 2100 Copenhagen, Denmark; Henrik.Ullum@regionh.dk

**Keywords:** *IL2RA*, CD25, multiple sclerosis, rs2104286, rs11256593, interleukin-2 receptor

## Abstract

Single nucleotide polymorphisms (SNPs) in or near the *IL2RA* gene, that encodes the interleukin-2 (IL-2) receptor α (CD25), are associated with increased risk of immune-mediated diseases including multiple sclerosis (MS). We investigated how the MS-associated *IL2RA* SNPs rs2104286 and rs11256593 are associated with CD25 expression on T cells ex vivo by multiparameter flow cytometry in paired genotype-selected healthy controls. We observed that MS-associated *IL2RA* SNPs rs2104286 and rs11256593 are associated with expression of CD25 in CD4^+^ but not CD8^+^ T cells. In CD4^+^ T cells, carriers of the risk genotype had a reduced frequency of CD25^+^ T_FH_1 cells (*p* = 0.001) and an increased frequency of CD25^+^ recent thymic emigrant cells (*p* = 0.006). Furthermore, carriers of the risk genotype had a reduced surface expression of CD25 in post-thymic expanded CD4^+^ T cells (CD31^−^CD45RA^+^), CD39^+^ T_Reg_ cells and in several non-follicular memory subsets. Our study found novel associations of MS-associated *IL2RA* SNPs on expression of CD25 in CD4^+^ T cell subsets. Insight into the associations of MS-associated *IL2RA* SNPs, as these new findings provide, offers a better understanding of CD25 variation in the immune system and can lead to new insights into how MS-associated SNPs contribute to development of MS.

## 1. Introduction

The cytokine interleukin-2 (IL-2) has an extensive array of effects that are central for the balance between immune tolerance and immunity [1,2]. The IL-2 receptor α chain (IL-2Rα), also known as CD25, specifically binds IL-2 and is a central component in the trimeric IL-2 receptor complex [1,3]. CD25 has a short cytoplasmic domain and is not involved in the downstream signal transduction [4]; instead CD25 delivers IL-2 to IL-2Rβ and γc in a stepwise assembly [5,6] and creates a high-affinity receptor complex for the low levels of IL-2 in vivo [3]. Responsiveness to IL-2 directly depends on the cell’s expression of CD25 as IL-2 signaling increases with its expression and correlates with the capacity to bind IL-2 in a stable IL-2/IL-2R complex [7,8].

Among T cells, there is heterogeneity in CD25 expression [7,8], with some T cells such as T regulatory (T_Reg_) cells constitutively expressing high levels [2,9]. However, on most cells expression of CD25 is inducible by T cell receptor activation and cytokine signals [3,7], including a positive IL-2 feed-back loop [10]. Heterogeneity in CD25 expression implies variability in the T cell’s ability to benefit from the shared pool of IL-2, resulting in a tug of war with T cells with the highest expression of CD25 as winners [8,11].

IL-2 signaling is important for the survival and suppressive capacity of T_Reg_ cells [1]. In addition, IL-2 modulates polarization of effector functions by inducing IFN-γ as well as receptors and transcription factors associated with a T helper 1 (T_H_1) committed lineage [12,13], and expression of CD25 is required for the optimal development of T_H_1 cells [14]. IL-2 is also crucial for the expression of GM-CSF in human T cells [15]. Conversely, expression of receptors and transcriptions factors associated with T_H_17 lineage commitment is inhibited by IL-2 signaling [12,13]. In a competing environment, T_Reg_ cells augment T_H_17 differentiation, possibly by depletion of IL-2 [16,17]. Additionally, decreased IL-2 signaling increases the differentiation of T follicular helper (T_FH_) cells [18,19], a CD4^+^ T cell subset able to provide specialized B cell help [20].

In CD8^+^ T cells, IL-2 signaling impacts the T cell’s fate decision between memory and effector differentiation [1]. Cells receiving prolonged and strong IL-2 signaling by maintaining high CD25 expression expand rapidly, show enhanced effector functions, and are driven towards a terminally differentiated cell fate and apoptosis [7,21,22]. Conversely, cells receiving less IL-2 signaling are not cytolytic and differentiate into long-lived functional memory cells associated with a central memory phenotype [7,22,23,24]. However, transient high-affinity IL-2 receptor signaling early after priming promotes development of highly functional effector memory cells that later give rise to long-lived central memory cells [25]. Furthermore, high-affinity IL-2 signaling is required for the ability of memory T cells to expand in a competing environment with other T cells and induce a potent secondary expansion [23,24].

Single nucleotide polymorphisms (SNPs) in or near the *IL2RA* gene, that encodes CD25, have been associated with increased risk of several immune-mediated diseases [26,27,28,29,30]—including multiple sclerosis (MS). MS is a common demyelinating neurological disease triggered by environmental factors in individuals with a complex genetic risk profile [31,32]. The pathogenesis of MS involves dysregulated T_Reg_ cells [33,34,35,36], increased T_FH_ activity [37], recruitment of proinflammatory CD4^+^ T cells to the CNS [31], accumulation of CD8^+^ T cells in CNS lesions [38], and increased concentration of soluble CD25 in sera [39].

The SNPs rs2104286 and rs11256593 in or near the *IL2RA* gene are associated with increased risk of developing MS [28,29,30]. The association between the SNP rs11256593 near the *IL2RA* gene and risk of MS has only recently been established in the MS replication chip study [29]. Previous studies of the MS-associated *IL2RA* SNP rs2104286 effects on immune cells have focused on a limited number of CD4^+^ T cell phenotypes. Carriers of the risk allele (T) for SNP rs2104286 were reported to have reduced IL-2 receptor signaling as measured by STAT5 phosphorylation [40], increased frequency of GM-CSF producing memory CD4^+^ T cells [15], increased frequency of CD25^+^ naïve T cells [41], and increased concentration of soluble CD25 [42]. Furthermore, studies in cell line models for helper and regulatory T cells have found that rs2104286 polymorphisms influence the activity of enhancer elements from the first intron in the *IL2RA* gene and the binding affinity of the transcription factor TFAP4 [43,44].

We aimed to investigate how CD25 expression is associated with MS-associated SNPs rs2104286 and rs11256593 in or near the *IL2RA* gene in human CD4^+^ and CD8^+^ T cell subsets ex vivo. We analyzed this in freshly isolated peripheral blood mononuclear cells (PBMC) from genotype-selected healthy controls by multiparameter flow cytometry using a paired experimental design allowing for the quantitative assessment of CD25 expression on a wide range of T cell subtypes. We confirm that homozygous carriers of the MS-associated *IL2RA* risk alleles have an increased frequency of CD25^+^ naïve CD4^+^ T cells and find that this difference is mainly observed in recent thymic emigrant cells. In addition, we report that homozygous carriers of the MS-associated *IL2RA* risk alleles have reduced CD25 expression on a wide range of memory CD4^+^ T cells and decreased frequency of CD25^+^ T_FH_1 cells.

## 2. Material and Methods

### 2.1. Study Participants

Study participants were recruited among 1000 healthy subjects in the Danish Blood Donor Cohort [45] who previously donated blood to the Danish Multiple Sclerosis Centers’ (DMSC) contribution to the International Multiple Sclerosis Genetics Consortium (IMSGC) replication chip study [29]. The study was conducted in accordance with the Declaration of Helsinki and the protocol was approved by the scientific Ethics Committee in the Capital Region of Denmark (H-15008896). All participants gave written informed consent for inclusion before they participated in the study. Participants were selected based on a comprehensive lifestyle questionnaire (translated from Swedish and used with permission from Karolinska Institute, Sweden) [46] and the SNPs rs2104286 and rs11256593. SNP rs11256593 is the strongest associated SNP in the *IL2RA* gene region and in linkage disequilibrium (LD) with the previously associated lead SNP rs2104286 [29].

Selected study participants were recruited using a paired study design where each pair consisted of a homozygous carrier of the risk allele (T) for both MS associated *IL2RA* SNPs rs11256593 and rs2104286, defined as the risk genotype, and a homozygous carrier of the protective allele (C) of both SNPs, defined as a protective genotype. In addition, each pair was of the same sex and had a maximum age difference of 5 years. All participants were of self-reported European ancestry in two generations and not regular smokers for one year prior to inclusion. They reported no family history of MS and did not have any known autoimmune diseases (including type 1 diabetes, rheumatoid arthritis, inflammatory bowel disease, thyroid disease, or systemic lupus erythematosus). Furthermore, pregnant healthy subjects and healthy subjects with previous cancer were not recruited. Study participants had no self-reported intake of systemic anti-inflammatory, immunosuppressive, or immunomodulatory medications.

By recruiting 25 pairs, each pair consisting of a homozygous carrier of the risk genotype and a homozygous carrier of the protective genotype, we had 80% power to detect a standardized difference of 0.55 between subjects carrying the protective and risk genotype in a paired comparison. In a previous study we found that the association of the *IL2RA* SNP rs2104286 on the plasma concentration of soluble CD25 showed a standardized difference of 1.5 [47]. By including 25 genotype matched pairs we had sufficient statistical power to detect *IL2RA* SNP associations less than 40% of the magnitude of the known association with soluble CD25 concentrations.

### 2.2. Genotyping

Genotyping was performed on Illumina’s MS replication custom bead chip and conducted by the International MS Genetics Consortium (IMSGC) [29].

### 2.3. Sample Collection and PBMC Isolation

Blood sampling and experimental procedures for each pair were performed on freshly prepared samples on the day of sample collection. Venous blood was collected in BD Vacutainer EDTA tubes (BD Bioscience, Plymouth, UK) from 1–2 fasting pairs between 7–9 a.m. Within 1 h the blood was diluted with phosphate buffered saline (PBS) (Life Technologies, Paisley, UK) containing 2mM EDTA (Thermo Fisher Scientific Baltics, Vilnius, Lithuania) and transferred to Leucosep tubes (Greiner Bio One; Frickenhausen, Germany), and the peripheral blood mononuclear cells (PBMCs) were isolated by density gradient centrifugation on Lymphoprep (Axis-Shield, Oslo, Norway). PBMCs were washed twice in PBS/EDTA.

### 2.4. Flow Cytometry Analysis

The flow cytometric analysis was performed on freshly isolated PBMC as a previous study showed that CD25 expression is affected by freeze–thaw procedures [41]. PBMCs were stained with fluorochrome-conjugated antibodies to identify blood CD4^+^ and CD8^+^ T cell subsets by flow cytometry. The staining procedure was conducted blinded to genotype and performed simultaneously for each pair. Prior to staining, FcR Blocking Reagent (Miltenyi Biotec, Bergisch Gladbach, Germany) was added to the PBMCs to prevent non-specific binding. We used the following monoclonal antibodies specific for: CD3 (PerCP/Cy5.5; UCHT1), CD3 (PE/Cy7; UCHT1), CD4 (APC/AF750; S3.5), CD8 (BV605; RPA-T8), CD25(PE; M-A251), CD127(BV421; A019D5), CD31 (BV605;WM59); CD39 (APC; A1); CD45RA (FITC;HI100), CXCR5 (APC;J252D4), PD-1 (BV605;EH12.2H7), CXCR3 (AF488;G025H7), CCR6 (PerCP/Cy5.5;G034E3), and CCR7 (AF647; G043H7). All antibodies were purchased from BioLegend (San Diego, CA, USA). The stained PBMCs were washed 3 times in cold PBS/2%FCS/0.1% NaN_3_ and data were acquired on a FACS Canto II 8 color flow cytometer (BD Biosciences, San Jose, CA, USA) aiming for 350,000 acquisitions.

### 2.5. Gating Strategy

**Figure 1:**
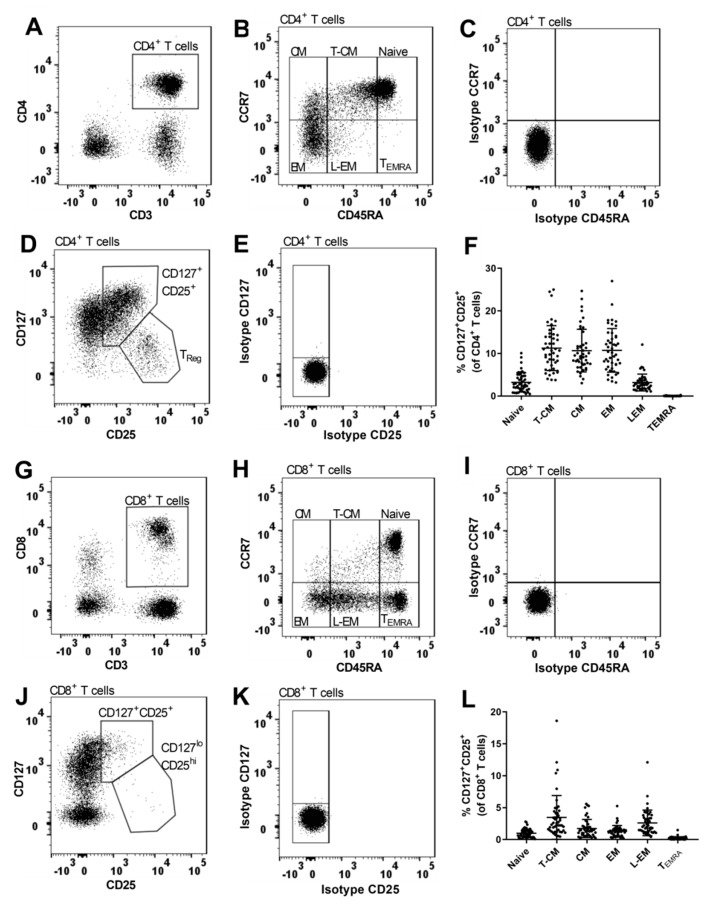
Gating strategy for CD4^+^ and CD8^+^ T cell differentiation. (**A**,**G**) Gating of CD4^+^ and CD8^+^ T cells. (**B**,**H**) Differential expression of CD45RA and CCR7 defines 6 subsets on CD4^+^ and CD8^+^ T cells: Naïve (CD45RA^hi^CCR7^+^), transitional central memory (T-CM) (CD45RA^lo-int^CCR7^+^), central memory (CM) (CD45RA^−^CCR7^+^), effector memory (EM) (CD45RA^−^CCR7^−^), late effector memory (L-EM) (CD45RA^lo-int^CCR7^−^), and T_EMRA_ (CD45RA^hi^CCR7^−^). CD45RA^hi^ (Naïve and T_EMRA_), and CD45RA^lo-int^(T-CM and L-EM) were gated on CD8^+^ T cells (H) and subsequently applied to CD4^+^ T cells (**B**). CCR7^+/−^ and CD45RA^−^ (CM and EM) were gated by use of matched isotype control. (**C**,**I**) Matched isotype controls for CCR7 and CD45RA on CD4^+^ and CD8^+^ T cells. (**D**,**J**) Differential expression of CD25 and CD127 defines two CD25^+^ populations on CD4^+^ and CD8^+^ T cells: CD127^+^CD25^+^ cells and T_Reg_ (CD127^lo^CD25^hi^) cells. (**E**,**K**) Matched isotype controls for CD25 and CD127 on CD4^+^ and CD8^+^ T cells. (**F**) The CD127^+^CD25^+^ gate is applied to the six CD4^+^ T cell subsets. Frequency of CD127^+^CD25^+^ naïve, T-CM, CM, EM, L-EM, and T_EMRA_ cells in relation to CD4^+^ T cells. (**L**) The CD127^+^CD25^+^ gate is applied to the six CD8^+^ T cell subsets. Frequency of CD127^+^CD25^+^ naïve, T-CM, CM, EM, L-EM, and T_EMRA_ cells in relation to CD8^+^ T cells.

Data analysis was performed blinded to genotype status using FlowJo software (Tree Star, Ashland, OR, USA). Gating strategies are shown in Figures 1, 3, 4 and 6. Matched isotype controls were applied where appropriate. Median fluorescence intensity (MFI) was used as a measure for surface expression of CD25 on CD25^+^ T cells (Figure 3D,E). Samples with a sub-optimal staining were excluded pair-wise before unblinding of genotype status. Thereby, we excluded 2 pairs that had poor staining of CD45RA; 3 pairs with poor staining of CD39 and CD31; 4 pairs with no usable sample for CD39/CD31.

### 2.6. Statistical Analysis

Statistical analysis was conducted with IBM SPSS Statistics 22 and GraphPad Prism 7. Normal distribution was determined by a Shapiro–Wilk test. Depending on normal distribution, all statistical analyses regarding genotype-phenotype associations were performed using a paired-sample t-test or a Wilcoxon Signed Rank test of samples collected the same day from matched pairs carrying either the risk or protective *IL2RA* alleles. The significance level was set at *p* ≤ 0.005 and *p*-values ≤ 0.05 were considered suggestive in accordance with recent recommendations [48].

## 3. Results

To identify genotype-phenotype associations between MS-associated gene variants in the *IL2RA* gene and changes in CD25 expression in T cells, healthy study participants were recruited based on genotype status for the MS associated *IL2RA* SNPs rs2104286 and rs11256593 and lifestyle information from a large cohort of healthy blood donors. Only healthy subjects were recruited as previous studies have suggested that immunological alterations in established MS may compromise the ability to demonstrate the biological associations of SNPs associated with MS risk [39,47]. Recruited participants were either homozygous carriers of the risk alleles (T) for both SNPs rs2104286 and rs11256593, defined as a risk genotype (TT) or homozygous carriers of the protective alleles (C) for both SNPs, defined as a protective genotype (CC). Two-hundred subjects met our recruitment criteria and were contacted. Approximately 100 responded and in total we recruited 50 healthy subjects who were paired with respect to sex, age and genotype status for experimental procedures and statistical analysis of 25 pairs (Table 1).

### 3.1. Expression of CD25 on Naïve and Memory CD4^+^ T Cell Populations

CD45RA and CCR7 expression were used to delineate CD4^+^ and CD8^+^ T cell differentiation [49]. In both CD4^+^ and CD8^+^ T cells, we observed a subpopulation with an intermediate expression of CD45RA and by the use of isotype control we found that it was not CD45RA^−^—a phenotypic characteristic associated with memory cells [49]. Thus, by differential expression of CD45RA and CCR7 we defined six subsets on CD4^+^ (Figure 1B) and CD8^+^ T cells (Figure 1H): Naïve (CD45RA^hi^CCR7^+^); transitional central memory (T-CM) (CD45RA^lo-int^CCR7^+^); central memory (CM) (CD45RA^−^CCR7^+^); effector memory (EM) (CD45RA^−^CCR7^−^); late-EM (L-EM) (CD45RA^lo-int^CCR7^−^), and terminally differentiated effector memory cells (T_EMRA_)(CD45RA^hi^CCR7^−^).

**Figure 2:**
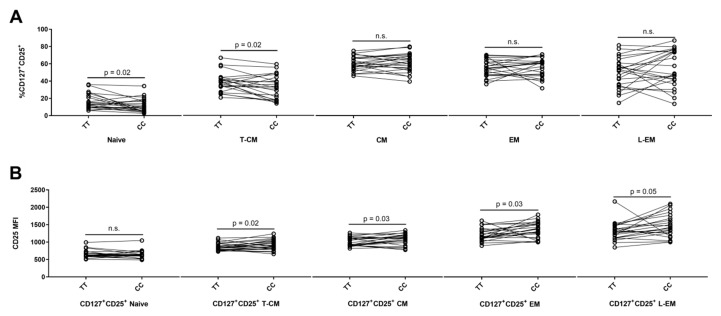
*IL2RA* genotype associations on differentiation of CD4^+^ T cells. Homozygous carriers of both MS-associated *IL2RA* SNPs, rs2104286 and rs11256593, are defined by TT for the risk genotype (N = 23) and CC for the protective genotype (N = 23). Gating strategy is outlined in Figure 1A–E. (**A**) Differences between TT carriers and CC carriers on frequency of CD127^+^CD25^+^ naïve (Paired t test, mean of difference = −4.6; 95% CI = −8.5 to −0.7), T-CM (Paired t test, mean of difference = −5.6; 95% CI = −10.4 to −0.9), CM (Paired t test, mean of difference = 0.6; 95% CI = −2.7 to 3.9), EM (Paired t test, mean of difference = 1.2; 95% CI = −4.0 to 6.4) and L-EM cells (Paired t test, mean of difference = 5.1; 95% CI = −3.6 to 13.8). (**B**) Differences between TT carriers (N = 23) and CC carriers (N = 23) on surface expression (MFI) of CD25 on CD127^+^CD25^+^ naïve (Paired t test, mean of difference = −16.9; 95% CI = −56.5 to 22.8), T-CM (Paired t test, mean of difference = 63.9; 95% CI = 13 to 114.8), CM (Paired t test, mean of difference = 61.5; 95% CI = 5.6 to 117.4), EM (Paired t test, mean of difference = 120; 95% CI = 13.4 to 226.7) and L-EM cells (Paired t test, mean of difference = 153.9; 95% CI = 0.9 to 306.8).

On CD4^+^ T cells, expression of CD25 and the IL-7 receptor α (CD127) identify two CD25^+^ CD4^+^ T cell populations: The non-regulatory CD127^+^CD25^+^ population and the regulatory CD127^lo^CD25^hi^ (T_Reg_) population [50,51] (Figure 1D). The CD127^+^CD25^+^ gate was applied to each of the six CD45RA/CCR7 subsets and we found that the highest frequency of CD127^+^CD25^+^ cells was in the T-CM, CM, and EM CD4^+^ T cell populations (Figure 1F). Carriers of the risk genotype (TT) had a suggestively higher frequency of CD127^+^CD25^+^ cells in naïve (*p* = 0.02) and T-CM (*p* = 0.02) populations compared to carriers of the protective genotype (CC) (Figure 2A). Conversely, carriers of the risk genotype (TT) had a suggestively reduced surface expression (MFI) of CD25 on CD127^+^CD25^+^ T cells in the T-CM (*p* = 0.02); CM (*p* = 0.03); EM (*p* = 0.03), and L-EM (*p* = 0.05) populations (Figure 2B). CD127^+^CD25^+^ T_EMRA_ cells only represent 0.018% (IQR: 0.008: 0.008–0.026%) of CD4^+^ T cells why these cells were excluded from statistical analysis.

### 3.2. The MS-Associated IL2RA Risk Genotype Has Few Associations with CD25 Expression on CD8^+^ T Cells

In CD8^+^ T cells, expression of CD127 and CD25 defined the same two populations as in CD4^+^ T cells (Figure 1J). The CD127^+^CD25^+^ population represented 8.4% (IQR: 6.0–12.1%). The CD127^+^CD25^+^ gate was applied to the six CD8^+^ T cell differentiation subsets (Figure 1H). We found that the T-CM population had the highest frequency of CD127^+^CD25^+^ cells among CD8^+^ T cells (Figure 1L) and that carriers of the risk genotype (TT) had a suggestively lower frequency of CD127^+^CD25^+^ T-CM cells (Table 2: *p* = 0.03). We observed no associations of the MS-associated *IL2RA* genotype with frequency or surface expression (MFI) of CD25 on the other CD127^+^CD25^+^ CD8^+^ T cell subsets (Table 2). In CD8^+^ T cells, the CD127^lo^CD25^hi^ population only represented 0.49% (IQR: 0.24–1.2%) and therefore excluded from statistical analysis.

### 3.3. MS-Associated IL2RA Risk Genotype Associations on CD31^+^ Recent Thymic Emigrant (RTE) and Non-RTE CD45RA^+^ CD4^+^ T Cells

**Figure 3:**
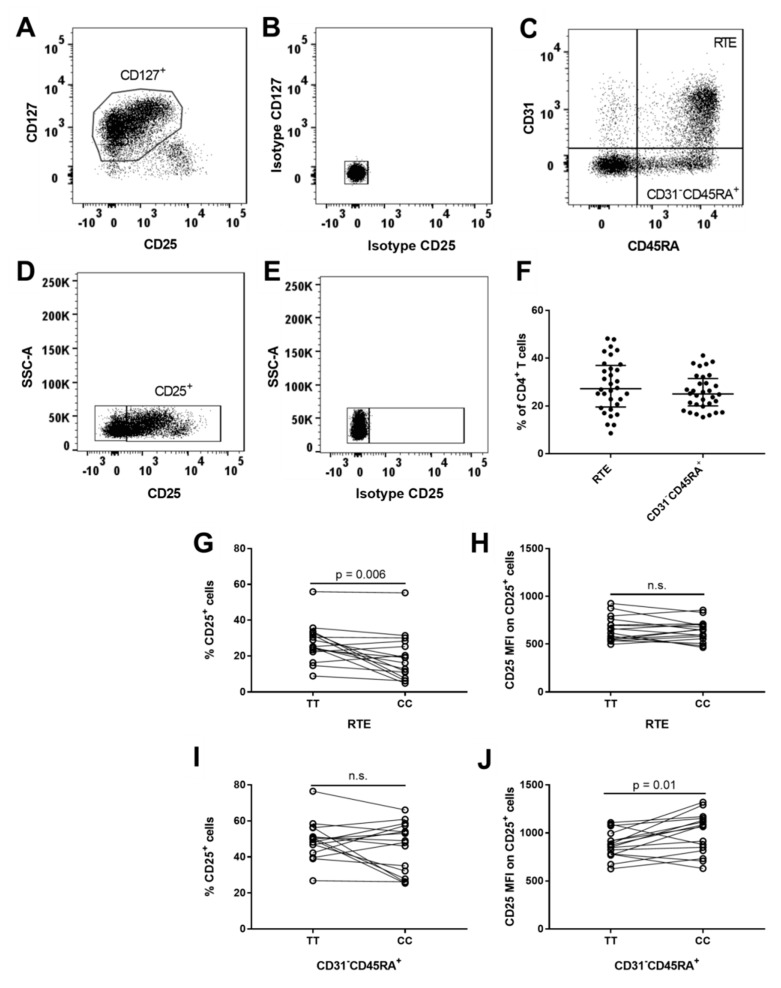
*IL2RA* genotype associations on recent thymic emigrants and CD31^−^CD45RA^+^ CD4^+^ T cells. (**A**) Gating of CD127^+^ cells on CD4^+^ T cells by use of matched isotype control. (**B**) Matched isotype control for CD25 and CD127 on CD4^+^ T cells. (**C**) Gating of recent thymic emigrants (RTE; CD31^+^CD45RA^+^) and CD31^−^CD45RA^+^ cells. RTE and CD31^−^CD45RA^+^ cells are gated from CD127^+^CD4^+^ T cells to exclude contamination from T_Reg_ cells. (**D**) Gating of CD25^+^ cells on CD4^+^ T cells by use of matched isotype control. (**E**) Matched isotype control for CD25. (**F**) Frequency of recent thymic emigrant (RTE; CD31^+^CD45RA^+^) cells and CD31^−^CD45RA^+^ cells in relation to CD4^+^ T cells. (**G**,**I**) Homozygous carriers of both MS-associated *IL2RA* SNPs rs2104286 and rs11256593 are defined by TT for the risk genotype and CC for the protective genotype. Differences between TT carriers (N = 16) and CC carriers (N = 16) on frequency of CD25^+^ cells in RTE (Paired t test, mean of difference = −8.0; 95% CI = −13.5 to −2.6) and CD31^−^CD45RA^+^ (Paired t test, mean of difference = −4.3; 95% CI = −11.4 to 2.7) T cells. (**H**,**J**) Differences between TT carriers (N = 16) and CC carriers (N = 16) on surface expression (MFI) of CD25 in CD25^+^RTE (Paired t test, mean of difference = −17.8; 95% CI = −67.1 to 31.6) and CD25^+^CD31^−^CD45RA^+^ (Paired t test, mean of difference = 120.3; 95% CI = 29.3 to 211.3) cells.

CD31 expression distinguishes CD31^+^CD45RA^+^ CD4^+^ T cells that are recent thymic emigrants (RTE) from CD31^−^CD45RA^+^ CD4^+^ T cells that have undergone post-thymic peripheral expansion [52]. In our study, the majority of CD45RA^+^ CD4^+^ T cells represented naïve and T-CM cells (Figure 1B). To exclude contamination from T_Reg_ cells, we gated RTE and CD31^−^CD45RA^+^ cells on CD127^+^ cells (Figure 3C). RTE cells comprised 27% (IQR: 20–37%) of CD4^+^ T cells (Figure 3F) and carriers of the risk genotype (TT) had a suggestively higher frequency of CD25^+^ RTE cells (*p* = 0.006) compared to carriers of the protective genotype (CC) (Figure 3G). However, we observed no difference in the expression (MFI) of CD25 on CD25^+^ RTE cells in relation to the *IL2RA* genotype (Figure 3H). In CD31^−^CD45RA^+^ cells, representing 25% (IQR: 20–32%) of CD4^+^ T cells (Figure 3F), we observed that carriers of the risk genotype (TT) had a suggestively decreased surface expression of CD25 on CD25^+^ CD31^−^CD45RA^+^ cells (*p* = 0.01) compared to carriers of the protective genotype (CC) (Figure 3J), but we found no *IL2RA* genotype associations with the frequency of CD25^+^ CD31^−^CD45RA^+^ cells (Figure 3I).

### 3.4. Lower Frequency of CD25^+^ T_FH_1 Cells in Carriers of the IL2RA Risk Genotype

**Figure 4:**
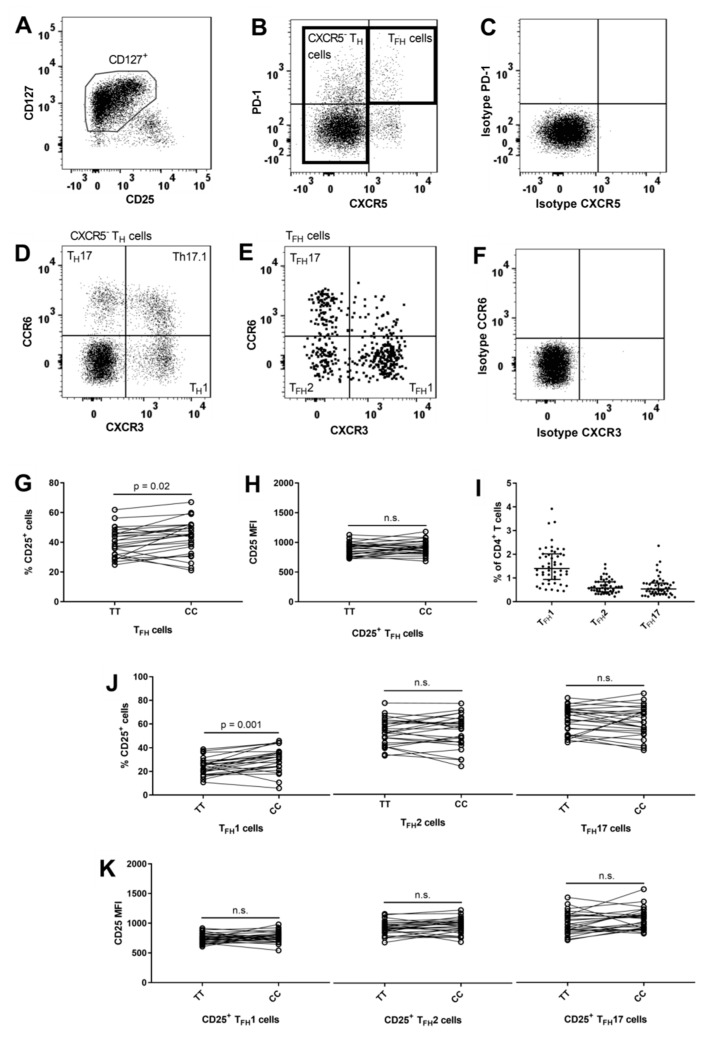
*IL2RA* genotype associations on CD4^+^ T follicular helper subsets. (**A**) Gating of CD127^+^ cells on CD4^+^ T cells by use of matched isotype control. (**B**) Gating of CXCR5^−^ cells, defined as non-follicular T helper (CXCR5^−^ T_H_) cells and T follicular helper (T_FH_) cells, defined by being PD-1^+^CXCR5^+^. Gating are performed on CD127^+^ CD4^+^ T cells to exclude contamination from T_Reg_ cells and by use of matched isotype control. (**C**) Matched isotype control for CXCR5 and PD-1 on CD4^+^ T cells. (**D**) On CXCR5^−^ T_H_ cells, differential expression of CXCR3 and CCR6 defines three subsets: T_H_1 (CXCR3^+^CCR6^−^), Th17.1 (CXCR3^+^CCR6^+^) and T_H_17 (CXCR3^−^CCR6^+^). Gating are performed by use matched isotype control. (**E**) On T_FH_ cells, differential expression of CXCR3 and CCR6 defines tree subsets: T_FH_1 (CXCR3^+^CCR6^−^), T_FH_2 (CXCR3^−^CCR6^−^) and T_FH_17 (CXCR3^−^CCR6^+^ T_FH_). Gating are performed by use matched isotype control. (**F**) Matched isotype control for CCR6 and CXCR3 on CD4^+^ T cells. (**G**,**H**) Homozygous carriers of both MS-associated *IL2RA* SNPs rs2104286 and rs11256593 are defined by TT for the risk genotype (N = 25) and CC for the protective genotype (N = 25). Difference between TT and CC carriers on the frequency of CD25^+^ cells in T_FH_ cells (Paired t test, mean of difference = 3.7; 95% CI = 0.7 to 6.7) and the surface expression (MFI) of CD25 on CD25^+^ T_FH_ cells (Paired t test, mean of difference = 20.7; 95% CI = −20 to 61.4). (**I**) Frequency of T_FH_1 (CXCR3^+^CCR6^−^ T_FH_), T_FH_2 (CXCR3^−^CCR6^−^ T_FH_) and T_FH_17 (CXCR3^−^CCR6^+^ T_FH_) cells in relation to CD4^+^ T cells. (**J**,**K**) Difference between carriers of the *IL2RA* risk genotype TT (N = 25) and the protective genotype CC (N = 25) on the frequency of CD25^+^ cells in T_FH_1 (Paired t test, mean of difference = 5.6; 95% CI = 2.4 to 8.7), T_FH_2 (Paired t test, mean of difference = 0.1; 95% CI = −3.2 to 3.4) and T_FH_17 (Paired t test, mean of difference = −1; 95% CI = −4.8 to 2.9) cells and the surface expression (MFI) of CD25 on CD25^+^T_FH_1 (Paired t test, mean of difference = 23.1; 95% CI = −20.6 to 66.8), CD25^+^T_FH_2 (Paired t test, mean of difference = 20.6; 95% CI = −22.5 to 63.8), and CD25^+^T_FH_17 (Paired t test, mean of difference = 63; 95% CI = −10.2 to 136.2) cells.

Blood T follicular helper (T_FH_) cells can be identified by surface expression of CXCR5 and PD-1 [20,53]. The PD-1^+^CXCR5^+^ (T_FH_) cells were gated on CD127^+^ cells to exclude contamination from T_Reg_ cells (Figure 4B) and comprised 3.1% (IQR: 2.0–3.8%) of CD4^+^ T cells. Carriers of the risk genotype (TT) had a suggestively lower frequency of CD25^+^ T_FH_ cells (*p* = 0.02) compared to carriers of the protective genotype (CC) (Figure 4G). Differential surface expression of chemokine receptors CXCR3 and CCR6 separates T_FH_1 (CCR6^−^CXCR3^+^), T_FH_2 (CCR6^−^CXCR3^−^), and T_FH_17 (CCR6^+^CXCR3^−^) cells [54] (Figure 4E). T_FH_1 cells made up the largest group of T_FH_ cells (Figure 4I) and we found that carriers of the risk genotype had a significantly lower frequency of CD25^+^ T_FH_1 cells (*p* = 0.001) while we observed no *IL2RA* genotype associations on the T_FH_2 and T_FH_17 cells (Figure 4J). We observed no *IL2RA* genotype associations with surface expression levels of CD25 on the CD25^+^ T_FH_ subsets (Figure 4K).

### 3.5. Reduced Surface Expression of CD25 on CXCR5^−^ T_H_1 and T_H_17 Cells in Carriers of the IL2RA Risk Genotype

**Figure 5:**
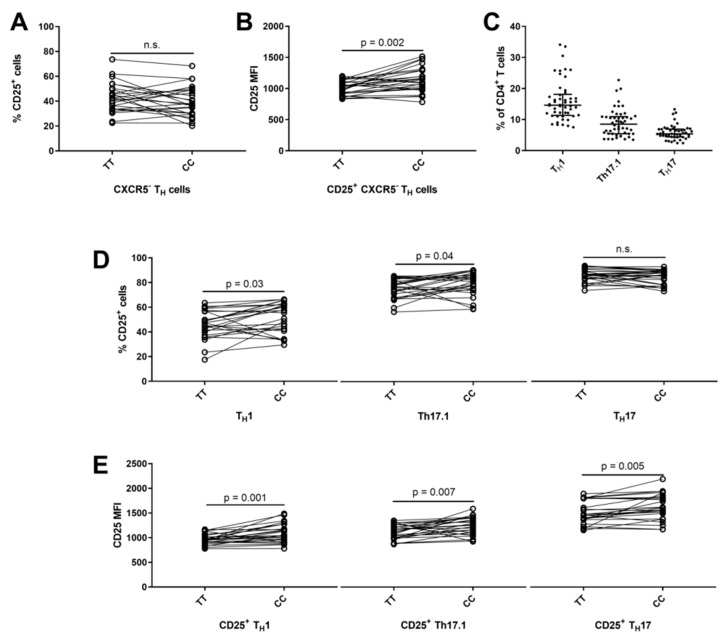
*IL2RA* genotype associations on CD4^+^ non-follicular T helper subsets. Non-follicular T helper (T_H_) subsets are defined by being CXCR5^−^ and gated from CD127^+^ cells to exclude contamination from T_Reg_ cells (Figure 4B). (**A**,**B**) Homozygous carriers of both MS-associated *IL2RA* SNPs rs2104286 and rs11256593 are defined by TT for the risk genotype and CC for the protective genotype. Difference between TT carriers (N = 25) and the protective genotype CC (N = 25) on the frequency of CD25^+^ cells in CXCR5^−^ T_H_ cells (Paired t test, mean of difference = −3.4; 95% CI = −7.9 to 1.2) and the surface expression (MFI) of CD25 on CD25^+^ CXCR5^−^ T_H_ cells (Paired t test, mean of difference = 121.7; 95% CI = 50.3 to 193.1). (**C**) Frequency of T_H_1 (CXCR3^+^CCR6^−^ CXCR5^−^ T_H_), Th17.1 (CXCR3^+^CCR6^+^ CXCR5^−^ T_H_) and T_H_17 (CXCR3^−^CCR6^+^ CXCR5^−^ T_H_) cells in relation to CD4^+^ T cells (gating outlined in Figure 4D). (**D**,**E**) Difference between TT carriers (N = 25) and CC carriers (N = 25) on the frequency of CD25^+^ cells in the T_H_1 (Paired t test, mean of difference = 5.5; 95% CI = 0.6 to 10.5), Th17.1 (Paired t test, mean of difference = 4.2; 95% CI = 0.1 to 8.3) and T_H_17 (Paired t test, mean of difference = −1.1; 95% CI = −3.4 to 1.2) subset and the surface expression (MFI) of CD25 on CD25^+^ T_H_1 (Paired t test, mean of difference = 120.2; 95% CI = 52.7 to 187.6), Th17.1 (Paired t test, mean of difference = 116.8; 95% CI = 35.6 to 198.1) and T_H_17 (Paired t test, mean of difference = 113.5; 95% CI = 38.7 to 188.4) cells.

Non-follicular helper T (T_H_) cells, defined as CXCR5^−^ and CD127^+^ (Figure 4B), comprised 80% (IQR: 77–82%) of CD4^+^ T cells. We observed that carriers of the risk genotype had a significantly lower surface expression of CD25 (*p* = 0.002) on CD4^+^ CD25^+^ CXCR5^−^ T_H_ cells (Figure 5B). Chemokine receptors CXCR3 and CCR6 can distinguish lineage commitment of CXCR5^−^ T_H_ cells and defines three subsets: T_H_1 (CCR6^−^CXCR3^+^) [55]; Th17.1 (CCR6^+^CXCR3^+^) [56,57] and T_H_17 (CCR6^+^CXCR3^−^) [56,58] (Figure 4D). Distribution of the three CXCR5^−^ T_H_ subsets can be seen in Figure 5C. Carriers of the risk genotype (TT) had a suggestively lower frequency of CD25^+^ Th17.1 cells (*p* = 0.04) and CD25^+^ T_H_1 cells (*p* = 0.03) compared to the protective genotype (Figure 5D). In addition, carriers of the risk genotype (TT) had a significant decrease in surface expression of CD25 on CD25^+^ T_H_1 (*p* = 0.001) and CD25^+^ T_H_17 cells (*p* = 0.005) and a suggestively lower surface expression of CD25 on CD25^+^ Th17.1 (*p* = 0.007) cells compared to carriers of the protective genotype (CC) (Figure 5E).

### 3.6. MS-Associated IL2RA Genotype Associations with Surface Expression of CD25 on T_Reg_ Cell Populations

**Figure 6:**
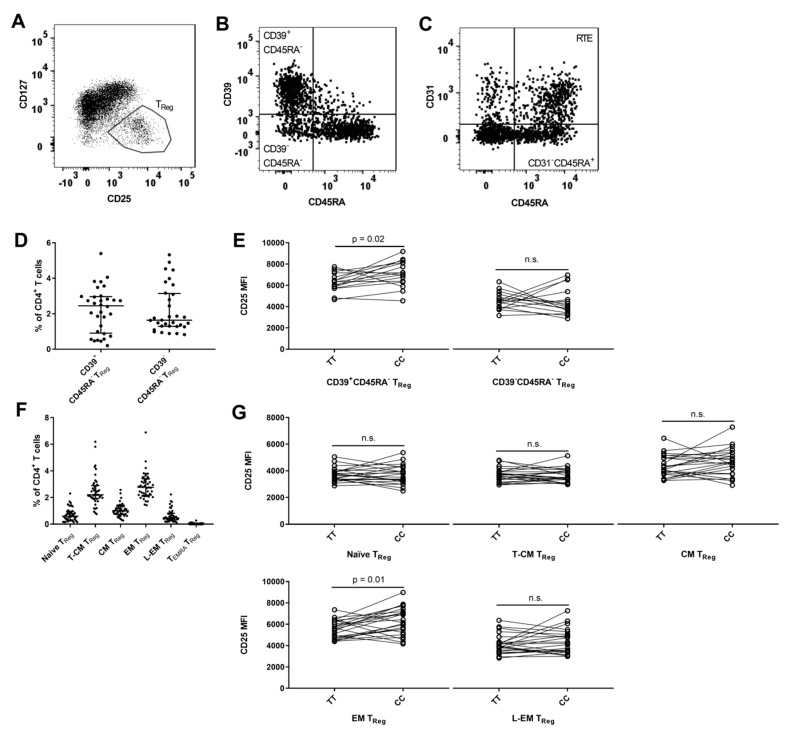
*IL2RA* genotype associations on CD4^+^ T_Reg_ subsets. (**A**) Gating of T_Reg_ cells, defined by being CD127^lo^CD25^hi^. (**B**) T_Reg_ cells are gated by expression of CD39 and CD45RA in two subsets: CD39^+^CD45RA^−^ (committed T_Reg_ cells) and CD39^−^CD45RA^−^. (**C**) T_Reg_ cells are gated by expression of CD31 and CD45RA in two subsets: Recent thymic emigrants (RTE; CD31^+^CD45RA^+^) and CD31^−^CD45RA^+^. (**D**) Frequency of CD39^+^CD45RA^−^ and CD39^−^CD45RA^−^ T_Reg_ in relation to CD4^+^ T cells. (**E**) Homozygous carriers of both MS-associated *IL2RA* SNPs rs2104286 and rs11256593 are defined by TT for the risk genotype and CC for the protective genotype. Differences between carriers of the risk genotype TT (N = 16) and carriers of the protective genotype CC (N = 16) on surface expression of CD25 (MFI) on CD39^+^CD45RA^−^ (Paired t test, mean of difference = 800.9; 95% CI = 175.2 to 1427) and CD39^−^CD45RA^−^ T_Reg_ (Paired t test, mean of difference = −110; 95% CI = −956.9 to 736.9) cells. (**F**) Frequency of naïve (CD45RA^hi^CCR7^+^), transitional central memory (T-CM) (CD45RA^lo-int^CCR7^+^), central memory (CM) (CD45RA^−^CCR7^+^), effector memory (EM) (CD45RA^−^CCR7^−^), late effector memory (L-EM) (CD45RA^lo-int^CCR7^+^) and T_EMRA_ (CD45RA^hi^CCR7^−^) cells with a T_Reg_ phenotype in relation to CD4^+^ T cells. Gating for CD4^+^ T cell differentiation are outlined in Figure 1B. (**G**) Differences between TT carriers (N = 23) and CC carriers (N = 23) on surface expression (MFI) of CD25 on T_Reg_ naïve (Paired t test, mean of difference = −25.9; 95% CI = −303.2 to 251.4), T-CM (Paired t test, mean of difference = −33; 95% CI = −262.1 to 196.1), CM (Paired t test, mean of difference = 278.1; 95% CI = −149.7 to 706), EM (Paired t test, mean of difference = 720.6; 95% CI = 181.5 to 1260) and L-EM (Paired t test, mean of difference = 240.6; 95% CI = −222.3 to 703.5) cells.

The T_Reg_ (CD127^lo^CD25^hi^) population comprised 7.4% (IQR: 6.6–8.6%) of CD4^+^ T cells. Expression of CD39 can identify a committed T_Reg_ cell [59] (Figure 6B). CD39^+^CD45RA^−^ T_Reg_ cells comprised 2.4% (IQR: 0.9–3.0%) of CD4^+^ T cells (Figure 6D) and carriers of the risk genotype (TT) had a suggestively lower surface expression of CD25 on CD39^+^CD45RA^−^ T_Reg_ cells (*p* = 0.02) (Figure 6E). As in non-regulatory CD4^+^ T cells, CD31 can identify CD45RA^+^ T_Reg_ cells that are RTE [36] (Figure 6C), a population that in our study comprised 1.5% (IQR: 0.7–2.0%) of CD4^+^ T cells. However, we found no difference between *IL2RA* genotypes in CD25 expression (data not shown).

Finally, we applied the T_Reg_ (CD127^lo^CD25^hi^) gate to each of the CD45RA/CCR7 subsets defined in Section 3.1 on CD4^+^ T cells (Figure 1B). We found that the highest frequency of T_Reg_ cells was in the EM (CD45RA^−^CCR7^−^) population in relation to CD4^+^ T cells (Figure 6F). Risk genotype (TT) carriers had a suggestively lower surface expression of CD25 on T_Reg_ cells with an EM phenotype (*p* = 0.01) (Figure 6G). Approximately no T_EMRA_ cells had a T_Reg_ phenotype and therefore this subset was excluded from statistical analysis.

## 4. Discussion

Expression of CD25 is central in T cells responsiveness to IL-2 [7,8]. There is great heterogeneity in CD25 expression on T cell subtypes [7,8], however, the contributions from MS-associated *IL2RA* genetic variants are poorly understood. We have systematically explored how the MS-associated SNPs rs2104286 and rs11256593, in or near the *IL2RA* gene, that encode CD25, are associated with CD25 expression on T cells by multiparameter flow cytometry in genotype-selected healthy subjects.

We found that carriers of the MS-associated *IL2RA* risk genotype (TT) had reduced surface expression of CD25 on CD25^+^CD127^+^CD4^+^ T cells that had undergone post-thymic peripheral expansion (CD31^−^CD45RA^+^) or had a memory phenotype, such as CXCR5^−^ T_H_ subsets or CD45RA^−/lo-int^ cells including EM-T_Reg_ and CD39^+^CD45RA^−^ T_Reg_ cells. Additionally, carriers of the risk genotype (TT) had a significantly lower frequency of CD25^+^ T_FH_1 cells and a suggestively lower frequency of CXCR5^−^ CD25^+^ T_H_1 and CD25^+^ Th17.1 cells. Finally, we confirmed that carriers of the risk genotype (TT) had an increased frequency of CD25^+^ CD45RA^+^ CD4^+^ T cells [15,41] and we found that this was due to an increased frequency of CD25^+^CD45RA^+^ CD4^+^ T cells that were recent thymic emigrants (CD31^+^).

Contrary to preceding studies [15,40,41], we only compared healthy subjects homozygous for the risk (T) or protective (C) allele, for both MS-associated SNPs rs11256593 and rs2104286, to eliminate gene-dosage effects on phenotypes previously observed for the rs2104286 SNP [15]. Furthermore, compared to preceding studies [15,40,41], we only included SNPs in or near the *IL2RA* gene associated with MS and did not include *IL2RA* SNPs associated with other autoimmune diseases which resulted in inclusion of the well-established SNP rs2104286 and the newly discovered SNP rs11256593 near the *IL2RA* gene from the MS-replication chip study [29]. In our cohort of a 1000 HCs only 8.3% were homozygous for the protective allele (C) for SNP rs2104286 and therefore we were not able to reproduce findings in a secondary cohort and it was only possible to determine the combined association of the MS-associated SNPs rs2104286 and rs11256593 and not the independent associations of the rs2104286 and rs11256593 SNPs on expression of CD25 without compromising the size and pairing of the genotype groups. A study determining the independent associations of rs2104286 and rs11256593 on CD25 expression are warranted. However, the selection cohort of study participants should exceed 1000 HCs as some *IL2RA* genotypes are rare.

Compared to previous studies, we applied a more in-depth phenotyping of T cell subsets including both CD8^+^ and CD4^+^ T cell subsets not previously studied [15,40,41]. In CD8^+^ T cells, we only observed a single suggestive difference although the number of individuals in each genotype group in CD8^+^ T cells was the same as for CD4^+^ T cells suggesting that MS-associated *IL2RA* SNPs rs2104286 and rs11256593 are associated with CD25 expression in CD4^+^, but not in CD8^+^ T cells. This is consistent with a previous study that observed that the rs2104286 SNP controls GM-CSF production in CD4^+^ but not in CD8^+^ T cells [15]. In addition, we found MS-associated *IL2RA* gene variants rs2104286 and rs11256593 are associated differently with CD25 expression within CD4^+^ T cell subgroups. In CD4^+^ T cells positive for either CD31 or CXCR5, MS-associated *IL2RA* variants were associated with the frequency of CD25^+^ cells but not the MFI of CD25. Contrary, in CD4^+^ T cells negative for either CD31 or CXCR5 we observed several associations between MFI of CD25 and MS-associated *IL2RA* gene variants as well as frequency of CD25^+^ cells. Our findings indicate that CD25 heterogeneity within CD4^+^ T cell subgroups are dependent on MS-associated *IL2RA* gene variants and emphasizes that cell subsets need to be clearly defined by several markers in studies of MS-associated *IL2RA* gene variants. In CD4^+^ T cells, use of too few cell markers for definition of cell subsets can lead to study of mixed effects and lead to inconclusive results.

IL-2 signaling increases with expression of CD25 [7,8] and a previous study by Cerosaletti et al. reported that homozygous carriers of MS-associated *IL2RA* risk allele for SNP rs2104286 had reduced IL-2 receptor signaling as assessed by STAT5 phosphorylation in CD25^hi^ and memory CD4^+^ T cells [40]. Thus, these results are consistent with our observation that homozygous carriers of MS-associated *IL2RA* risk allele rs2104286 and rs11256593 have decreased MFI of CD25 on T_Reg_ cells with an EM phenotype or CD39^+^ and CD4^+^CD127^+^CD25^+^ T cell subsets being CD45RA^−/lo^ or expressing CXCR3 and/or CCR6, markers preferentially expressed on memory CD4^+^ T cells [49]. Cerosaletti et al. did examine if the observed lower STAT5 phosphorylation was due to decreased surface expression of CD25 but contrary to our results found no difference between carriers of the risk and protective allele for SNP rs2104286 [40]. However, Cerosaletti et al. performed stainings on cells from not paired HCs that had undergone a freeze–thaw process while we stained freshly isolated PBMCs from paired HCs. A previous study found that CD4^+^ memory cells undergone a freeze–thaw process due to cryopreservation had between 28–62% lower expression of CD25 compared to CD4^+^ memory cells analyzed in fresh whole blood [41] which can explain the discrepancy between the studies. In addition, pairing of the samples as performed in our study reduces day-to-day variation in staining and is more able to identify associations between MS-associated *IL2RA* gene variants and CD25 expression in cell subsets where cell frequency is age dependent. Finally, the discrepancy between Cerosaletti et al. and our study can be due to difference in cell markers applied to identifying cell subsets and subsequent gating strategy [40].

Analyzing freshly isolated PBMCs limits our study to a steady-state setting and cannot address the extent to which MS-associated *IL2RA* gene variants rs2104286 and rs11256593 influence T cells IL-2 signaling and effector responses upon activation without compromising our studies ability to identify genotype associations on ex vivo CD25 surface expression and frequency. CD25 expression correlate with T cells responsiveness to IL-2 [7,8], a central cytokine for T_Reg_ cells ability to sustain suppressive function and modulates effector responses and T helper lineage commitment [1]. It is a possibility that in a competing immune environment the reduced expression of CD25 in carriers of the *IL2RA* risk genotype may inhibit the CD4^+^ T cells’ ability to benefit from the shared pool of IL-2 and as a result promote CD4^+^ T cells normally inhibited by IL-2 such as T_H_17 and T_FH_ cells [12,13,18,19], and inhibit cells normally promoted by IL-2 such as T_H_1 cells and T_Reg_ cells [1,12,13]. Furthermore, it is well-established that risk allele carriers of the MS-associated *IL2RA* SNP rs2104286 have increased concentration of soluble CD25 in sera [42]. Studies have found that soluble CD25 inhibit the action of IL-2 on T cells [39] and may have a functional implication for modulation of T cell responses in MS [60]. In relation to our observation of decreased surface bound CD25, it might be a possibility that carriers of the *IL2RA* risk genotype have more unstable CD25 that leads to the lower levels of surface bound CD25 and higher levels of soluble CD25 and this exerts a dual effect on IL-2 responsiveness in CD4^+^ T cells. As mentioned, the present study is not able to address these questions, however future studies should address how the reduced CD25 expression observed in carriers of the MS-associated *IL2RA* risk allele affects CD4^+^ T cells IL-2 responsiveness and whether this changes T helper cells effector functions and T_Reg_ cells suppressive capacity and if there is a dual effect with soluble CD25. Interestingly, in carriers of the *IL2RA* MS-associated risk allele we observed a significant reduction in the frequency of CD25^+^ T_FH_1 cells and a suggestive reduction in the frequency of CD25^+^ T_H_1 and Th17.1 cells indicating a possible shift in effector functions. Furthermore, such studies could also provide new insight to if and how MS-associated *IL2RA* SNPs contribute to the impaired suppressive capacity of CD39^+^ T_Reg_ cells [33], the reduced frequency of CXCR5^+^ T_FH_1 cells [37] and the promotion of pro-inflammatory CD4^+^ T cells observed in MS patients [31].

In summary, we found that MS-associated *IL2RA* SNPs rs2104286 and rs11256593 are associated with CD25 expression on CD4^+^, but not in CD8^+^ T cells. In CD4^+^ T cells, MS-associated *IL2RA* gene variants are associated differently within subsets where carriers of the MS-associated risk allele (TT) show decreased surface expression of CD25 on memory CD127^+^CD25^+^ and T_Reg_ subsets, increased frequency of CD25^+^ RTE T cells and decreased frequency of CD25^+^ T_FH_1 cells. These changes in CD25 expression may influence the cells IL-2 signaling thus affecting CD4^+^ T cell differentiation and T_Reg_ cells suppressive activity.

## Figures and Tables

**Table 1:** Demographic characteristics for the 50 healthy subjects included in the study. A risk genotype (TT) is defined as a homozygous carrier of the risk allele (T) for both MS-associated *IL2RA* SNPs rs2104286 and rs11256593. A protective genotype (CC) is defined as a homozygous carrier of the protective allele (C) for both MS-associated *IL2RA* SNPs rs2104286 and rs11256593.

	Risk Genotype Group	Protective Genotype Group
	(TT)	(CC)
N	25	25
Mean age (yr)	46.7	47.0
Age range (yr)	24–68	24–70
Male:female ratio	9:16	9:16

**Table 2:** *IL2RA* genotype effects on the CD127^+^CD25^+^ population in relation to CD8^+^ T cell differentiation. A risk genotype (TT) is defined as a homozygous carrier of the risk allele (T) for both MS-associated *IL2RA* SNPs rs2104286 and rs11256593. A protective genotype (CC) is defined as a homozygous carrier of the protective allele (C) for both MS-associated *IL2RA* SNPs rs2104286 and rs11256593.

Subset *	N_Pair_	Median Frequency of CD127^+^CD25^+^ Cells		Wilcoxon Signed Rank Test	Median MFI ** of CD25 on CD127^+^CD25^+^ Cells		Wilcoxon Signed Rank Test
		CC	TT		CC	TT	
Naive	23	3.4	2.7	n.s.	728	610	n.s.
T-CM	23	34.3	25.2	*0.03*	972	866	n.s.
CM	23	34.7	35.8	n.s.	1337	1240	n.s.
EM	23	12.3	11.8	n.s.	1296	1269	n.s.
L-EM	23	9.6	7.7	n.s.	892	827	n.s.
T_EMRA_	23	0.9	0.7	n.s.	745	788	n.s.

* Subset abbreviations: T-CM = Transitional central memory; CM = Central memory; EM = Effector memory; L-EM = Late-EM; T_EMRA_ = Terminally differentiated effector memory. ** MFI = median fluorescence intensity. *p* value depicting a trend for association is indicated in italics.

## References

[1] Boyman O., Sprent J. (2012). The role of interleukin-2 during homeostasis and activation of the immune system. Nat. Rev. Immunol..

[2] Malek T.R., Castro I. (2010). Interleukin-2 Receptor Signaling: At the Interface between Tolerance and Immunity. Immunity.

[3] Kim H.P., Imbert J., Leonard W.J. (2006). Both integrated and differential regulation of components of the IL-2/IL-2 receptor system. Cytokine Growth Factor Rev..

[4] Lin J.-X., Leonard W.J. (1997). Signaling from the IL-2 receptor to the nucleus. Cytokine Growth Factor Rev..

[5] Wang X., Rickert M., Garcia K.C. (2005). Structure of the quaternary complex of interleukin-2 with its alpha, beta, and gammac receptors. Science.

[6] Stauber D.J., Debler E.W., Horton P.A., Smith K.A., Wilson I.A. (2006). Crystal structure of the IL-2 signaling complex: Paradigm for a heterotrimeric cytokine receptor. Proc. Natl. Acad. Sci. USA.

[7] Kalia V., Sarkar S., Subramaniam S., Haining W.N., Smith K.A., Ahmed R. (2010). Prolonged Interleukin-2Rα Expression on Virus-Specific CD8+ T Cells Favors Terminal-Effector Differentiation In Vivo. Immunity.

[8] Feinerman O., Jentsch G., Tkach K.E., Coward J.W., Hathorn M.M., Sneddon M.W., Emonet T., Smith K.A., Altan-Bonnet G. (2010). Single-cell quantification of IL-2 response by effector and regulatory T cells reveals critical plasticity in immune response. Mol. Syst. Biol..

[9] Baecher-Allan C., Brown J.A., Freeman G.J., Hafler D.A. (2001). CD4+CD25high regulatory cells in human peripheral blood. J. Immunol..

[10] Kim H.-P., Kelly J., Leonard W.J. (2001). The Basis for IL-2-Induced IL-2 Receptor α Chain Gene Regulation: Importance of Two Widely Separated IL-2 Response Elements. Immunity.

[11] Bielekova B. (2013). Daclizumab therapy for multiple sclerosis. Neurotherapeutics.

[12] Liao W., Lin J.-X., Wang L., Li P., Leonard W.J. (2011). Modulation of cytokine receptors by IL-2 broadly regulates differentiation into helper T cell lineages. Nat. Immunol..

[13] Laurence A., Tato C.M., Davidson T.S., Kanno Y., Chen Z., Yao Z., Blank R.B., Meylan F., Siegel R., Hennighausen L. (2007). Interleukin-2 Signaling via STAT5 Constrains T Helper 17 Cell Generation. Immunity.

[14] Pepper M., Pagán A.J., Igyártó B.Z., Taylor J.J., Jenkins M.K. (2011). Opposing Signals from the Bcl6 Transcription Factor and the Interleukin-2 Receptor Generate T Helper 1 Central and Effector Memory Cells. Immunity.

[15] Hartmann F.J., Khademi M., Aram J., Ammann S., Kockum I., Constantinescu C., Gran B., Piehl F., Olsson T., Codarri L. (2014). Multiple sclerosis-associated IL2RA polymorphism controls GM-CSF production in human TH cells. Nat. Commun..

[16] Chen Y., Haines C.J., Gutcher I., Hochweller K., Blumenschein W.M., McClanahan T., Hämmerling G., Li M.O., Cua D.J., McGeachy M.J. (2011). Foxp3+ Regulatory T Cells Promote T Helper 17 Cell Development In Vivo through Regulation of Interleukin-2. Immunity.

[17] Pandiyan P., Conti H.R., Zheng L., Peterson A.C., Mathern D.R., Hernández-Santos N., Edgerton M., Gaffen S.L., Lenardo M.J. (2011). CD4+CD25+Foxp3+ Regulatory T Cells Promote Th17 Cells In Vitro and Enhance Host Resistance in Mouse Candida albicans Th17 Cell Infection Model. Immunity.

[18] Johnston R.J., Choi Y.S., Diamond J.A., Yang J.A., Crotty S. (2012). STAT5 is a potent negative regulator of TFH cell differentiation. J. Exp. Med..

[19] Choi Y.S., Kageyama R., Eto D., Escobar T.C., Johnston R.J., Monticelli L., Lao C., Crotty S. (2011). ICOS Receptor Instructs T Follicular Helper Cell versus Effector Cell Differentiation via Induction of the Transcriptional Repressor Bcl6. Immunity.

[20] Schmitt N., Bentebibel S.-E., Ueno H. (2014). Phenotype and functions of memory Tfh cells in human blood. Trends Immunol..

[21] Obar J.J., Molloy M.J., Jellison E.R., Stoklasek T.A., Zhang W., Usherwood E.J., Lefrancois L. (2010). CD4+ T cell regulation of CD25 expression controls development of short-lived effector CD8+ T cells in primary and secondary responses. Proc. Natl. Acad. Sci. USA.

[22] Pipkin M.E., Sacks J.A., Cruz-Guilloty F., Lichtenheld M.G., Bevan M.J., Rao A. (2010). Interleukin-2 and Inflammation Induce Distinct Transcriptional Programs that Promote the Differentiation of Effector Cytolytic T Cells. Immunity.

[23] Williams M.A., Tyznik A.J., Bevan M.J. (2006). Interleukin-2 signals during priming are required for secondary expansion of CD8+ memory T cells. Nature.

[24] Bachmann M.F., Wolint P., Walton S., Schwarz K., Oxenius A. (2007). Differential role of IL-2R signaling for CD8+ T cell responses in acute and chronic viral infections. Eur. J. Immunol..

[25] Castro I., Dee M.J., Malek T.R. (2012). Transient Enhanced IL-2R Signaling Early during Priming Rapidly Amplifies Development of Functional CD8+ T Effector-Memory Cells. J. Immunol..

[26] Lowe C.E., Cooper J.D., Brusko T., Walker N.M., Smyth D.J., Bailey R., Bourget K., Plagnol V., Field S., Atkinson M. (2007). Large-scale genetic fine mapping and genotype-phenotype associations implicate polymorphism in the IL2RA region in type 1 diabetes. Nat. Genet..

[27] Okada Y., Wu D., Trynka G., Raj T., Terao C., Ikari K., Kochi Y., Ohmura K., Suzuki A., Yoshida S. (2014). Genetics of rheumatoid arthritis contributes to biology and drug discovery. Nature.

[28] Beecham A.H., Patsopoulos N.A., Xifara D.K., Davis M.F., Kemppinen A., Cotsapas C., Shah T.S., Spencer C., Booth D., Goris A. (2013). Analysis of immune-related loci identifies 48 new susceptibility variants for multiple sclerosis. Nat. Genet..

[29] Patsopoulos N., Baranzini S.E., Santaniello A., Shoostari P., Cotsapas C., Wong G., Beecham A.H., James T., Replogle J., International Multiple Sclerosis Genetics Consorti (2017). The Multiple Sclerosis Genomic Map: Role of peripheral immune cells and resident microglia in susceptibility. BioRxiv.

[30] Sawcer S., Hellenthal G., Pirinen M., Spencer C.C.A., Patsopoulos N.A., Moutsianas L., Dilthey A., Su Z., Freeman C., Hunt S.E. (2011). Genetic risk and a primary role for cell-mediated immune mechanisms in multiple sclerosis. Nature.

[31] Nylander A., Hafler D.A. (2012). Multiple sclerosis. J. Clin. Investig..

[32] Baecher-Allan C., Kaskow B.J., Weiner H.L. (2018). Multiple Sclerosis: Mechanisms and Immunotherapy. Neuron.

[33] Fletcher J.M., Lonergan R., Costelloe L., Kinsella K., Moran B., O’Farrelly C., Tubridy N., Mills K.H.G. (2009). CD39+Foxp3+ regulatory T Cells suppress pathogenic Th17 cells and are impaired in multiple sclerosis. J. Immunol..

[34] Viglietta V., Baecher-Allan C., Weiner H.L., Hafler D.A. (2004). Loss of functional suppression by CD4+CD25+ regulatory T cells in patients with multiple sclerosis. J. Exp. Med..

[35] Carbone F., De Rosa V., Carrieri P.B., Montella S., Bruzzese D., Porcellini A., Procaccini C., la Cava A., Matarese G. (2014). Regulatory T cell proliferative potential is impaired in human autoimmune disease. Nat. Med..

[36] Haas J., Fritzsching B., Trübswetter P., Korporal M., Milkova L., Fritz B., Vobis D., Krammer P.H., Suri-Payer E., Wildemann B. (2007). Prevalence of newly generated naive regulatory T cells (Treg) is critical for Treg suppressive function and determines Treg dysfunction in multiple sclerosis. J. Immunol..

[37] Christensen J.R., Börnsen L., Ratzer R., Piehl F., Khademi M., Olsson T., Sørensen P.S., Sellebjerg F. (2013). Systemic Inflammation in Progressive Multiple Sclerosis Involves Follicular T-Helper, Th17- and Activated B-Cells and Correlates with Progression. PLoS ONE.

[38] Salou M., Nicol B., Garcia A., Laplaud D.-A. (2015). Involvement of CD8+ T Cells in Multiple Sclerosis. Front. Immunol..

[39] Maier L.M., Anderson D.E., Severson C.A., Baecher-Allan C., Healy B., Liu D.V., Wittrup K.D., de Jager P.L., Hafler D.A. (2009). Soluble IL-2RA levels in multiple sclerosis subjects and the effect of soluble IL-2RA on immune responses. J. Immunol..

[40] Cerosaletti K., Schneider A., Schwedhelm K., Frank I., Tatum M., Wei S., Whalen E., Greenbaum C., Kita M., Buckner J. (2013). Multiple autoimmune-associated variants confer decreased IL-2R signaling in CD4+ CD25(hi) T cells of type 1 diabetic and multiple sclerosis patients. PLoS ONE.

[41] Dendrou C.A., Plagnol V., Fung E., Yang J.H.M., Downes K., Cooper J.D., Nutland S., Coleman G., Himsworth M., Hardy M. (2009). Cell-specific protein phenotypes for the autoimmune locus IL2RA using a genotype-selectable human bioresource. Nat. Genet..

[42] Maier L.M., Lowe C.E., Cooper J., Downes K., Anderson D.E., Severson C., Clark P.M., Healy B., Walker N., Aubin C. (2009). IL2RA genetic heterogeneity in multiple sclerosis and type 1 diabetes susceptibility and soluble interleukin-2 receptor production. PLoS Genet..

[43] Butter F., Davison L., Viturawong T., Scheibe M., Vermeulen M., Todd J.A., Mann M. (2012). Proteome-Wide Analysis of Disease-Associated SNPs That Show Allele-Specific Transcription Factor Binding. PLoS Genet..

[44] Schwartz A.M., Demin D.E., Vorontsov I.E., Kasyanov A.S., Putlyaeva L.V., Tatosyan K.A., Kulakovskiy I.V., Kuprash D.V. (2017). Multiple single nucleotide polymorphisms in the first intron of the IL2RA gene affect transcription factor binding and enhancer activity. Gene.

[45] Pedersen O.B., Erikstrup C., Kotzé S.R., Sørensen E., Petersen M.S., Grau K., Ullum H. (2012). The Danish Blood Donor Study: A large, prospective cohort and biobank for medical research. Vox Sang..

[46] Hedström A.K., Hillert J., Olsson T., Alfredsson L. (2014). Alcohol as a Modifiable Lifestyle Factor Affecting Multiple Sclerosis Risk. JAMA Neurol..

[47] Buhelt S., Ratzer R.L., Christensen J.R., Börnsen L., Sellebjerg F., Søndergaard H.B. (2017). Relationship between soluble CD25 and gene expression in healthy individuals and patients with multiple sclerosis. Cytokine.

[48] Benjamin D.J., Berger J.O., Johannesson M., Nosek B.A., Wagenmakers E.-J., Berk R., Bollen K.A., Brembs B., Brown L., Camerer C. (2018). Redefine statistical significance. Nat. Hum. Behav..

[49] Mahnke Y.D., Brodie T.M., Sallusto F., Roederer M., Lugli E. (2013). The who’s who of T-cell differentiation: Human memory T-cell subsets. Eur. J. Immunol..

[50] Seddiki N., Santner-Nanan B., Martinson J., Zaunders J., Sasson S., Landay A., Solomon M., Selby W., Alexander S.I., Nanan R. (2006). Expression of interleukin (IL)-2 and IL-7 receptors discriminates between human regulatory and activated T cells. J. Exp. Med..

[51] Liu W., Putnam A.L., Xu-Yu Z., Szot G.L., Lee M.R., Zhu S., Gottlieb P.A., Kapranov P., Gingeras T.R., Groth B.F.D. (2006). CD127 expression inversely correlates with FoxP3 and suppressive function of human CD4+ T reg cells. J. Exp. Med..

[52] Kimmig S., Przybylski G.K., Schmidt C.A., Laurisch K., Möwes B., Radbruch A., Thiel A. (2002). Two subsets of naive T helper cells with distinct T cell receptor excision circle content in human adult peripheral blood. J. Exp. Med..

[53] Shi J., Hou S., Fang Q., Liu X., Liu X., Qi H. (2018). PD-1 Controls Follicular T Helper Cell Positioning and Function. Immunity.

[54] Morita R., Schmitt N., Bentebibel S.-E., Ranganathan R., Bourdery L., Zurawski G., Foucat E., Dullaers M., Oh S., Sabzghabaei N. (2011). Human Blood CXCR5+CD4+ T Cells Are Counterparts of T Follicular Cells and Contain Specific Subsets that Differentially Support Antibody Secretion. Immunity.

[55] Bonecchi R., Bianchi G., Bordignon P.P., D’Ambrosio D., Lang R., Borsatti A., Sozzani S., Allavena P., Gray P.A., Mantovani A. (1998). Differential expression of chemokine receptors and chemotactic responsiveness of type 1 T helper cells (Th1s) and Th2s. J. Exp. Med..

[56] Acosta-Rodriguez E.V., Rivino L., Geginat J., Jarrossay D., Gattorno M., Lanzavecchia A., Sallusto F., Napolitani G. (2007). Surface phenotype and antigenic specificity of human interleukin 17–producing T helper memory cells. Nat. Immunol..

[57] Paulissen S.M.J., Van Hamburg J.P., Dankers W., Lubberts E. (2015). The role and modulation of CCR6+ Th17 cell populations in rheumatoid arthritis. Cytokine.

[58] Singh S.P., Zhang H.H., Foley J.F., Hedrick M.N., Farber J.M. (2008). Human T cells that are able to produce IL-17 express the chemokine receptor CCR6. J. Immunol..

[59] Gu J., Ni X., Pan X., Lu H., Lu Y., Zhao J., Zheng S.G., Hippen K.L., Wang X., Lu L. (2017). Human CD39hi regulatory T cells present stronger stability and function under inflammatory conditions. Cell. Mol. Immunol..

[60] Russell S.E., Moore A.C., Fallon P.G., Walsh P.T. (2012). Soluble IL-2Rα (sCD25) exacerbates autoimmunity and enhances the development of Th17 responses in mice. PLoS ONE.

